# Diagnostic test accuracy of an automated device for the MALDI target preparation for microbial identification

**DOI:** 10.1007/s10096-022-04531-3

**Published:** 2022-12-05

**Authors:** Abdessalam Cherkaoui, Arnaud Riat, Gesuele Renzi, Adrien Fischer, Jacques Schrenzel

**Affiliations:** 1 Bacteriology Laboratory, Division of Laboratory Medicine, Department of Diagnostics, Geneva University Hospitals, 4 Rue Gabrielle-Perret-Gentil, 1205 Geneva, Switzerland; 2 Faculty of Medicine, Geneva, Switzerland; 3 Genomic Research Laboratory, Division of Infectious Diseases, Department of Medicine, Geneva University Hospitals and Faculty of Medicine, Geneva, Switzerland

**Keywords:** MALDI-TOF/MS, Automation, WASPLab®, Copan Colibrí™

## Abstract

The objective of this study was to evaluate the performance of the Copan Colibrí™ against the manual preparation of the MALDI targets. We analyzed 416 (31 different species) non-duplicate strains covering the most important species identified in clinical routine. We also assessed the intra-strain repeatability between the comparable methods. We then analyzed the performance of this new method after implementation in routine on 12,253 aerobic bacterial isolates and yeasts, encompassing a total of 42 different species. Among the 416 strains analyzed, 6.3% (26/416) and 10.8% (45/416) had a score value < 2 when processed by the Colibri™ and manual method, respectively. Only 5.9% (9/152) of the Gram positive rods and cocci had a score values < 2 by the Colibri™ versus 20.4% (31/152) by the manual method. We confirmed that this relative superiority observed for the Colibri™ was due primarily in the use of the formic acid protocol. For the Gram-negative bacteria, the results of both methods were comparable; 6.6% (17/256) and 4.7% (12/256) had a score value < 2 by the Colibri™ and the manual method, respectively. After implementation in routine, the results according to the Biotyper score cut-off values were distributed as follows: < 1.70: 2.5% (304/12,253), 1.70–1.79: 1.9% (227/12,253), 1.80–1.89: 3.1% (377/12,253), 1.90–1.99: 6.7% (825/12,253), and ≥ 2: 85.9% (10,520/12,253). The Colibrí™ coupled to MALDI-TOF/MS revealed good performances and higher intra-strain repeatability as compared to the manual preparation of the MALDI targets.

**Supplementary Information:**

The online version contains supplementary material available at 10.1007/s10096-022-04531-3.

## Introduction

Over the last decade, a large number of studies have assessed the performance of Matrix-Assisted Laser Desorption Time-of-Flight Mass Spectrometry (MALDI-TOF/MS) for the identification of bacteria, mycobacteria, yeasts, and molds in comparison with the well-established conventional diagnostic methods [1–6]. Accurate and rapid identification of microorganisms by MALDI-TOF/MS is contributing to swiftly support treatment decisions, especially when the identification of the pathogen is unexpected [7–9]. Hence, the real improvement of the turn-around times by MALDI-TOF/MS in conjunction with antibiotic stewardship could enable stopping unnecessary antibiotics and likely improving patient outcomes [10]. The implementation of total laboratory automation (TLA) in clinical microbiology has leveraged the advancement brought by MALDI-TOF/MS. Specifically by facilitating additional structural and organizational changes [11–14]. Copan’s TLA has recently integrated an automated device (Colibrí™) that can reproducibly prepare the MALDI target for microbial identification as well as a standardized bacterial inoculum for AST. The Colibrí™ integrates a pipetting system that permits the picking of the specific colonies defined by the technologists on the WASPLab screen (reading step), the transfer of the bacterial cells on the MALDI target, and the deposition of the matrix. These automated steps can improve the workflow and laboratory organization while simultaneously reducing the handling errors and ensuring proper documentation.

The main objective of the present study was to assess the accuracy and the diagnostic performances of the Colibrí™ coupled to MALDI-TOF/MS for the routine identification of a large number of clinical aerobic bacteria and yeasts.

## Material and methods

**Fig. 1:**
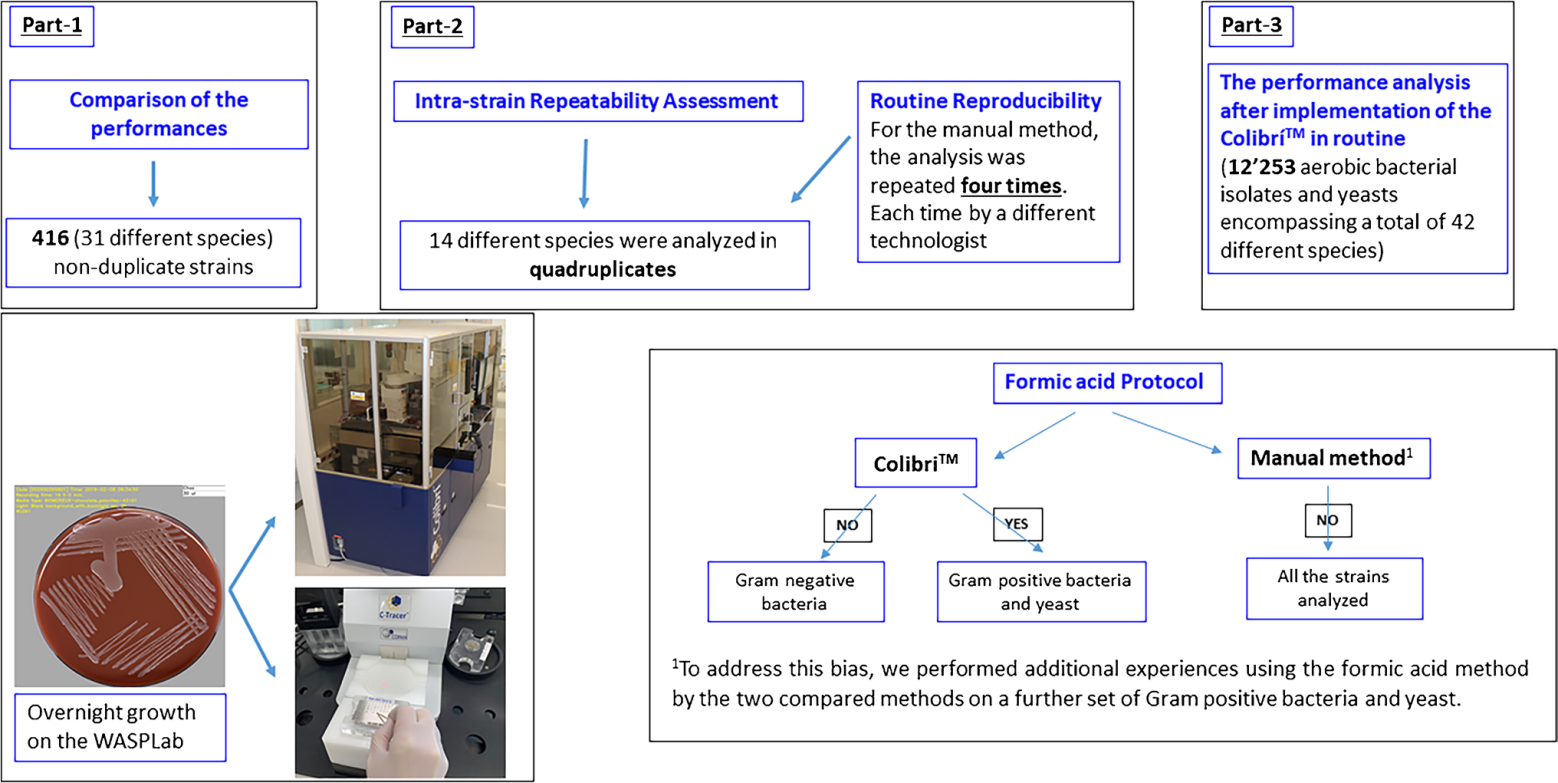
Comparative study design (the Colibrí.™ against the manual method for MALDI target preparation)

Figure 1 gave an overview of the present study design. We first evaluated the performance of the Colibrí™ coupled to MALDI-TOF/MS (MBT Compass 4.1, Bruker Daltonics, Bremen, Germany) against the manual preparation of MALDI target on 416 (31 different species) non-duplicate strains covering the most important species identified in clinical routine. Primarily the strains were subcultured on Columbia blood agar and chocolate blood agar using the WASPLab. The MALDI targets for the two compared methods were prepared using the same culture media plates by selecting colonies after overnight growth. Each isolate was analyzed in only one position on the MALDI target plate.

To assess the intra-strain repeatability between the Colibrí™ and the manual preparation of MALDI target, 14 strains from 14 different species were analyzed in quadruplicates on the MALDI target plates using overnight growth cultures on the WASPLab. For the manual method, the MALDI targets were independently prepared for each of the 14 strains by four experienced technologists, thereby assessing the routine reproducibility. The same culture media plates (Columbia blood agar and chocolate blood agar for *Haemophilus influenza*e) were used for the two compared methods.

After implementation of the Colibrí™ in routine, we analyzed the performance of this new method on 12,253 aerobic bacterial isolates and yeasts encompassing a total of 42 different species. This corresponds to all routine specimens sequentially referred to the bacteriology laboratory of Geneva University Hospitals between May and October 2021. In compliance with the routine procedures for the identification by MALDI-TOF/MS and to maintain an optimal turnaround time, the preparation of the MALDI target was performed by the Colibrí™ from different culture media used in our laboratory, according to the specimen’s type (e.g., chromogenic media, chocolate agar, CNA Columbia agar, MacConkey agar, and Columbia agar) without any subculture on a non-selective media. Each isolate was analyzed in only one position of the MALDI target plate.

The Colibrí™ proposes two protocols with or without an extraction step using formic acid. Hence, different protocols can be applied on different spots. The technologist defines the appropriate protocol by spot on the WASPLab during the reading step.

In this study, we used the Colibri™ protocol with formic acid for the Gram-positive bacteria and yeast, and without formic acid for the Gram-negative bacteria. In contrast, the manual method did not apply formic acid on any strain analyzed in compliance with our routine procedures. To address this bias, we performed additional experiences using the formic acid method by the two compared methods on a further set of Gram-positive bacteria and yeast.

We used the Biotyper scoring and the processing protocols according to the manufacturers’ instructions.

## Results

Table 1 depicts the performances of the Colibri™ and manual preparation of MALDI target according to the Biotyper score values. Among the 416 (31 different species) strains analyzed, 6.3% (26/416) and 10.8% (45/416) had a score value < 2 by the Colibri™ and manual method, respectively. For the Gram-negative bacteria, the results of both methods were comparable; 6.6% (17/256) and 4.7% (12/256) had a score value < 2 by the Colibri™ and manual method, respectively (Table 2 and Table S1). For Gram-positive rods and cocci, 5.9% (9/152) had a score values < 2 by the Colibri™ versus 20.4% (31/152) by the manual method (Table 2 and Table S1). Considering that we did not use formic acid for the manual method, we hypothesized that the differences observed in the score values for the Gram-positive rods and cocci between the Colibri™ and the manual method could be related to the formic acid protocol used with the Colibri. To test this hypothesis, we performed additional experiments using the formic acid in the two compared methods (i.e., the Colibri™ and the manual method) on an additional set of Gram-positive bacteria and yeasts (Table S2). The conclusion is that the difference observed for Gram-positive rods and cocci in Table 2 and Table S1 was related to the formic acid because no significant difference was observed when both methods used the formic acid.

**Table 1:** Performances of the Colibri™ and manual preparation of MALDI target according to Biotyper score values

Number total of strains analyzed = 416	Biotyper score	Nb of strains	Percentage
Colibri™	** < 1.7**	1	0.2
	**1.7– < 2**	25	6.0
	** ≥ 2**	390	93.8
Manual	** < 1.7**	5	1.2
	**1.7– < 2**	40	9.6
	** ≥ 2**	371	89.2

**Table 2:** Details of strains with a Biotyper score values < 2

(a) GRAM −	Biotyper score	Nb of strains	Percentage
Colibri™	** < 1.7**	1	5.9
	**1.7– < 2**	16	94.1
Manual	** < 1.7**	0	
	**1.7– < 2**	12	100
(b) GRAM +	Biotyper score	Nb of strains	Percentage
Colibri™	** < 1.7**	0	
	**1.7– < 2**	9	100
Manual	** < 1.7**	5	16.1
	**1.7– < 2**	26	83.9
(c) *Candida*	Biotyper score	Nb of strains	Percentage
Colibri™	** < 1.7**	0	
	**1.7– < 2**	0	
Manual	** < 1.7**	0	
	**1.7– < 2**	2	100

The eight *Candida albicans* strains analyzed had a score values > 2 by the Colibri™ (Table 2 and Table S1).

**Fig. 2:**
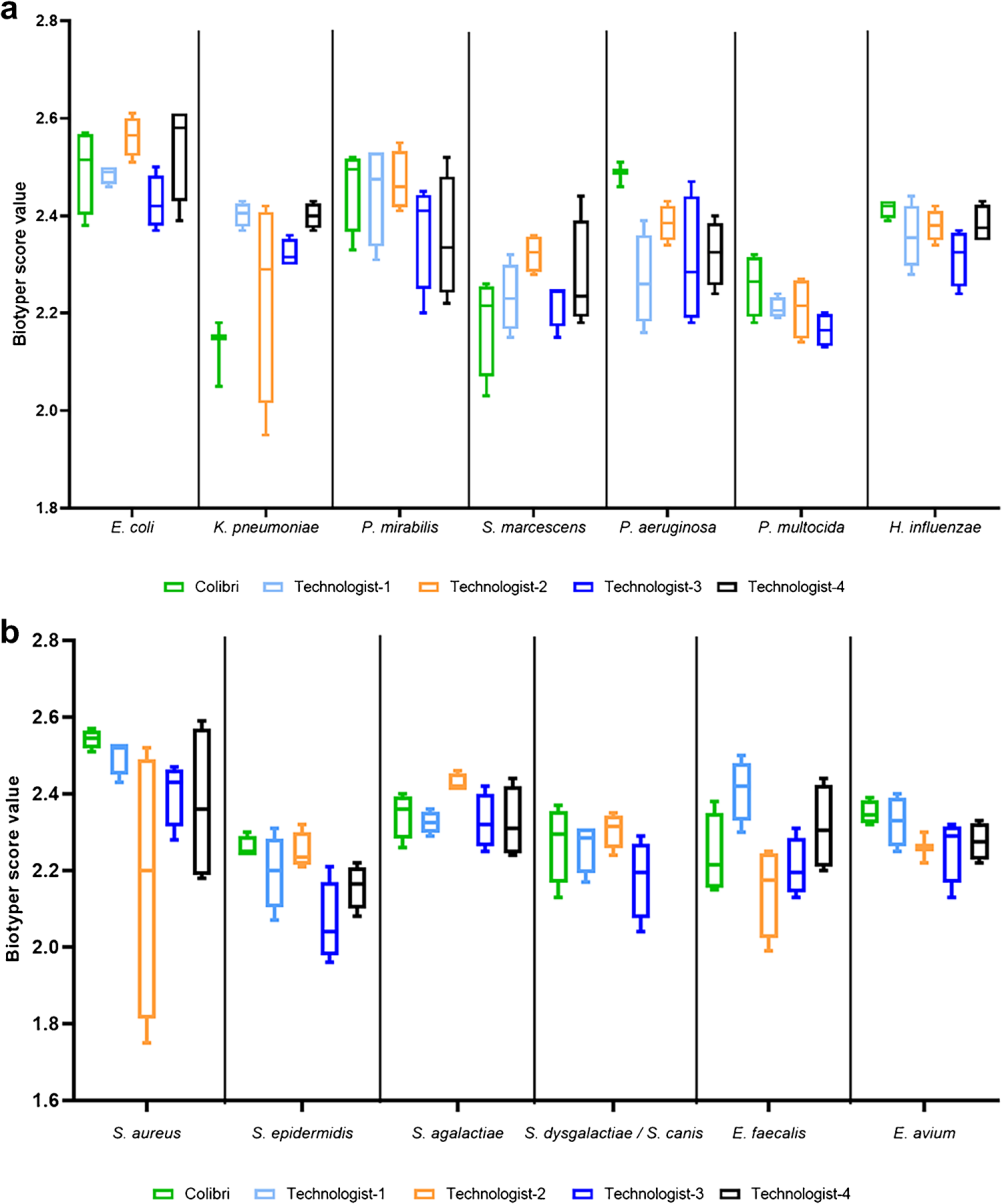
Box-plots show the intra-strain repeatability between the Colibrí™ and the manual preparation of MALDI targets. **a** Gram-negative bacteria. **b** Gram-positive bacteria. Each strain was analyzed in quadruplicate on the MALDI target plates using overnight growth culture. For the manual method, the MALDI targets were independently performed for each of these 14 strains by four experienced technologists, thereby evaluating the reproducibility

The analysis of the intra-strain repeatability between the Colibri™ and the manual preparation of MALDI target showed that the score values of all the strains processed by the Colibri™ were > 2. However, the inter-technologist reproducibility showed a large variation in the score values for identical isolates (Fig. 2a and b). The quadruplicates of all strains analyzed presented a score value > 2 using the Colibri™. Among the 56 spots (4 by strain) prepared manually, 4 (7.1%) gave a score value < 2.

After implementation of the Colibri™ in our routine workflow, a total of 3904 Enterobacterales, 2009 non-fermenting Gram-negative bacilli, 120 Gram-negative coccobacilli, 2806 staphylococci, 1245 enterococci, 785 streptococci, 458 Gram-positive aerobic bacilli, and 926 yeasts were sequentially assessed in this study. The Colibrí™ coupled to MALDI-TOF/MS identified 18 different genera encompassing 42 different species. Of the 12,253 isolates, 97.5% (11,494/12,253) gave an acceptable identification to the genus level (scores of ≥ 1.7). High-confidence species identification (scores of ≥ 2.0) was observed for 85.9% (10,520/12,253) of the isolates. In more details, 93.1% (3635/3904), 78.5% (1577/2009), 84.4% (2368/2806), 95.6% (1190/1245), and 63.9% (592/926) of the analyzed isolates gave a high confidence species identification (scores of ≥ 2.0) for Enterobacterales, non-fermenting Gram-negative bacilli, staphylococci, enterococci, and yeasts, respectively. Among the remaining isolates, 6.7% (825/12,253) yielded a score ranging between 1.90 and 1.99. Importantly, most were observed among *Staphylococcus epidermidis* (14.9%—123/825), *Stenotrophomonas maltophilia* (7.5%—62/825), and *Candida albicans* (21.7%—179/825). Regarding isolates reported with low confidence scores, 3.1% (377/12,253) and 1.9% (227/12,253) yielded scores ranging between 1.80–1.89 and 1.70–1.79, respectively. Finally, 2.5% (304/12,253) failed to report acceptable confidence scores (< 1.70) and had to be manually sub-cultivated. Among these isolates, the highest proportion originated from *Pseudomonas putida* (11.8%—36/304), *Candida albicans* (10.2%—31/304), and *Staphylococcus epidermidis* (7.6%—23/304) (Table 3).

**Table 3:** Performances of the Copan Colibrí™ coupled to MALDI-TOF/MS for microbial identification after implementation in routine

	Organism (no. of isolates tested)	Number of isolates (%) according to Biotyper score				
		< 1.7	1.7–1.79	1.8–1.89	1.9–1.99	> 2
Enterobacteriales	*Escherichia coli* (944)	11 (1.2)	6 (0.6)	16 (1.7)	31 (3.3)	880 (93.2)
	*Klebsiella aerogenes* (154)	0 (0)	0 (0)	2 (1.3)	4 (2.6)	148 (96.1)
	*Klebsiella oxytoca* (141)	4 (2.8)	5 (3.5)	2 (1.4)	7 (5.0)	123 (87.2)
	*Klebsiella pneumoniae* (963)	10 (1)	8 (0.8)	22 (2.3)	37 (3.8)	886 (92.0)
	*Klebsiella variicola* (93)	0 (0)	1 (1.1)	1 (1.1)	4 (4.3)	87 (93.5)
	*Morganella morganii* (223)	1 (0.4)	0 (0)	0 (0)	1 (0.4)	221 (99.1)
	*Proteus mirabilis* (448)	0 (0)	1 (0.2)	3 (0.7)	5 (1.1)	439 (98.0)
	*Citrobacter freundii* (248)	1 (0.4)	3 (1.2)	3 (1.2)	6 (2.4)	235 (94.8)
	*Citrobacter koseri* (111)	1 (0.9)	1 (0.9)	3 (2.7)	0 (0)	106 (95.5)
	*Serratia marcescens* (81)	2 (2.5)	1 (1.2)	1 (1.2)	4 (4.9)	73 (90.1)
	*Enterobacter asburiae* (64)	0 (0)	0 (0)	0 (0)	3 (4.7)	61 (95.3)
	*Enterobacter cloacae* (434)	10 (2.3)	8 (1.8)	12 (2.8)	28 (6.5)	376 (86.6)
Non-fermenting Gram-negative bacilli	*Pseudomonas aeruginosa* (1004)	21 (2.1)	10 (1)	12 (1.2)	37 (3.7)	924 (92.0)
	*Pseudomonas monteilii* (118)	18 (15.3)	9 (7.6)	8 (6.8)	14 (11.9)	69 (58.5)
	*Pseudomonas nitroreducens* (138)	16 (11.6)	7 (5.1)	14 (10.1)	15 (10.9)	86 (62.3)
	*Pseudomonas putida* (168)	36 (21.4)	21 (12.5)	20 (11.9)	20 (11.9)	71 (42.3)
	*Achromobacter xylosoxidans* (92)	4 (4.3)	2 (2.2)	10 (10.9)	16 (17.4)	60 (65.2)
	*Acinetobacter baumannii* (50)	2 (4.0)	0 (0)	1 (2)	3 (6)	44 (88.0)
	*Acinetobacter pittii* (50)	0 (0)	0 (0)	1 (2)	5 (10)	44 (88.0)
	*Stenotrophomonas maltophilia* (389)	14 (3.6)	8 (2.1)	26 (6.7)	62 (15.9)	279 (71.7)
*Haemophilus*	*Haemophilus influenzae* (61)	1 (1.6)	1 (1.6)	0 (0)	4 (6.6)	55 (90.2)
	*Haemophilus parainfluenzae* (59)	2 (3.4)	3 (5.1)	2 (3.4)	5 (8.5)	47 (79.7)
*Staphylococcus*	*Staphylococcus aureus* (968)	13 (1.3)	4 (0.4)	12 (1.2)	22 (2.3)	917 (94.7)
	*Staphylococcus capitis* (98)	3 (3.1)	3 (3.1)	1 (1)	12 (12.2)	79 (80.6)
	*Staphylococcus epidermidis* (1118)	23 (2.1)	30 (2.7)	54 (4.8)	123 (11.0)	888 (79.4)
	*Staphylococcus haemolyticus* (307)	13 (4.2)	8 (2.6)	18 (5.9)	30 (9.8)	238 (77.5)
	*Staphylococcus hominis* (114)	2 (1.8)	0 (0)	3 (2.6)	7 (6.1)	102 (89.5)
	*Staphylococcus lugdunensis* (146)	12 (8.2)	6 (4.1)	9 (6.2)	19 (13.0)	100 (68.5)
	*Staphylococcus saprophyticus* (55)	3 (5.5)	2 (3.6)	1 (1.8)	5 (9.1)	44 (80.0)
*Enterococcus*	*Enterococcus faecalis* (1018)	8 (0.8)	10 (1)	10 (1)	14 (1.4)	976 (95.9)
	*Enterococcus faecium* (227)	3 (1.3)	4 (1.8)	1 (0.4)	5 (2.2)	214 (94.3)
*Streptococcus*	*Streptococcus agalactiae* (541)	4 (0.7)	5 (0.9)	8 (1.5)	13 (2.4)	511 (94.5)
	*Streptococcus anginosus* (109)	4 (3.7)	4 (3.7)	4 (3.7)	9 (8.3)	88 (80.7)
	*Streptococcus oralis* (54)	6 (11.1)	2 (3.7)	1 (1.9)	1 (1.9)	44 (81.5)
	*Streptococcus mitis* (47)	2 (4.3)	2 (4.3)	6 (12.8)	6 (12.8)	31 (66.0)
	*Streptococcus pyogenes* (34)	1 (2.9)	0 (0)	0 (0)	0 (0)	33 (97.1)
*Gardnerella*	*Gardnerella vaginalis* (246)	12 (4.9)	7 (2.8)	10 (4.1)	29 (11.8)	188 (76.4)
*Corynebacterium*	*Corynebacterium amycolatum* (52)	2 (3.8)	2 (3.8)	1 (1.9)	3 (5.8)	44 (84.6)
	*Corynebacterium aurimucosum* (104)	3 (2.9)	4 (3.8)	10 (9.6)	22 (21.2)	65 (62.5)
	*Corynebacterium striatum* (56)	2 (3.6)	0 (0)	0 (0)	2 (3.6)	52 (92.9)
*Candida*	*Candida albicans* (829)	31 (3.7)	34 (4.1)	63 (7.6)	179 (21.6)	522 (63.0)
	*Candida glabrata* (97)	3 (3.1)	5 (5.2)	6 (6.2)	13 (13.4)	70 (72.2)
	Total (12,253)	304 (2.5)	227 (1.9)	377 (3.1)	825 (6.7)	10,520 (85.9)

## Discussion

The manual preparation of the MALDI target for microbial identification (ID) remains a tedious and error-prone task especially with the ever-increasing number of ID processed daily in diagnostic laboratories. In addition, the quality of the spot (i.e., the delivery of the adequate amount of microorganisms) on the MALDI target is highly operator-dependent and can affect the performance of the ID score as well as the laboratory workflow, by requiring re-test. While the use of formic acid, when deemed necessary, can improve the yield of identification, its execution further complicates the preparation of the MALDI target and complicates the laboratory workflow. The Colibrí™ enables a smooth integration of formic acid in the process without further complicating the workflow. The intra-strain repeatability and the inter-technologist reproducibility assessment revealed that the Colibri™ made good performances and higher intra-strain repeatability.

A comparative analysis on the 416 clinical strains revealed that all the Enterobacteriales strains were identified with a score value > 2 using the Colibri™. That was not the case for non-fermenting Gram-negative bacilli. Among *Pseudomonas monteilii*, *Pseudomonas putida*, and *Achromobacter xylosoxidans*, strains analyzed 66.7 (16/24) displayed a score value < 2 using the Colibri™ and required a re-test. We did not apply the formic acid protocol on these strains. When observing a score value < 2, we routinely re-test the sample, using the same media plate, because the Colibri™ uses precisely the pickpoint previously defined by the technologist during the reading step (WASPLab). Thus, if the position of the pickpoint is slightly offset from the microorganism’s colony, the score value can be affected. Our second step is to re-test the sample, after subculture on Columbia blood agar if the score value remains < 2.

Since the implementation of MALDI-TOF/MS in microbiology laboratories, a large number of studies reported that the approach is efficient, rapid, inexpensive, and highly accurate for the identification of the overwhelming majority of bacteria and yeasts [15–17]. While it allows the correct identification to the species level for a high percentage of microorganisms, the use of the conservative scoring system defined by the manufacturer decreases its diagnostic performance by rejecting some correctly identified microorganisms. As highlighted in various studies, adjusting this scoring system, relying on carefully validated studies, could significantly improve the number of correct identifications to the species level. The results of the study conducted by Scott et al. [18] suggest that it is unusual for a MALDI-TOF/MS to identify microorganisms incorrectly with a high score.

Overall, MALDI-TOF/MS has become an invaluable diagnostic tool in microbiology laboratories. The positive impact of the MALDI-TOF/MS to reduce the turn-around times (TAT) was also thoroughly evaluated. Tan et al. [19] reported that MALDI-TOF/MS enabled the reduction of TATs by an average of 1.45 days in comparison with the traditional phenotypic methods used for the identification.

The TLA has leveraged the improvements brought by MALDI-TOF/MS. In our previous study, we defined the shortest incubation times on the WASPLab for reliable MALDI-TOF/MS-based species for positive blood cultures. The percentage of bacterial strains identified with a score value > 2 were 73% (380/520) and 85% (440/520) after 4 h and 6 h of incubation of the culture media plates on the WASPLab, respectively [20]. The percentage of accurate identification (score value > 2) reached 100% (460/460) after 8 h of incubation when excluding *Corynebacterium* and *Candida* spp. from the analysis. This study established that the WASPLab coupled to MALDI-TOF/MS reduces significantly the TAT for positive blood cultures. Therefore, the implementation of the Colibri™ has substantially improved the workflow by reducing hands-on time required for manual preparation of the MALDI targets.

## Conclusions

The manual preparation of the MALDI target brings some degree of errors through, for example, the staggered positioning of the spots on the target. Moreover, the inter-technologist reproducibility revealed large variations in score values for a series of identical isolates. The Colibri™ contributes to a more reliable quality of the spot, improved traceability, and smooth integration of formic acid in the process, wherever needed. The Colibrí™ coupled to MALDI-TOF/MS revealed good performances and higher intra-strain repeatability as compared to the manual preparation of the MALDI targets.

## Supplementary Information

Below is the link to the electronic supplementary material.

Supplementary file1 (PDF 1420 KB)

## Data Availability

Not applicable.

## References

[1] Barberis C, Almuzara M, Join-Lambert O, Ramirez MS, Famiglietti A, Vay C (2014). Comparison of the Bruker MALDI-TOF mass spectrometry system and conventional phenotypic methods for identification of Gram-positive rods. PLoS ONE.

[2] Gaillot O, Blondiaux N, Loiez C, Wallet F, Lemaitre N, Herwegh S, Courcol RJ (2011). Cost-effectiveness of switch to matrix-assisted laser desorption ionization-time of flight mass spectrometry for routine bacterial identification. J Clin Microbiol.

[3] Torres-Sangiao E, Leal Rodriguez C, Garcia-Riestra C (2021). Application and perspectives of MALDI-TOF mass spectrometry in clinical microbiology laboratories. Microorganisms.

[4] Chen XF, Hou X, Xiao M, Zhang L, Cheng JW, Zhou ML, Huang JJ, Zhang JJ, Xu YC, Hsueh PR (2021). Matrix-assisted laser desorption/ionization time of flight mass spectrometry (MALDI-TOF MS) analysis for the identification of pathogenic microorganisms: a review. Microorganisms.

[5] Brown-Elliott BA, Fritsche TR, Olson BJ, Vasireddy S, Vasireddy R, Iakhiaeva E, Alame D, Wallace RJ, Branda JA (2019). Comparison of two commercial matrix-assisted laser desorption/ionization-time of flight mass spectrometry (MALDI-TOF MS) systems for identification of nontuberculous mycobacteria. Am J Clin Pathol.

[6] Cherkaoui A, Hibbs J, Emonet S, Tangomo M, Girard M, Francois P, Schrenzel J (2010). Comparison of two matrix-assisted laser desorption ionization-time of flight mass spectrometry methods with conventional phenotypic identification for routine identification of bacteria to the species level. J Clin Microbiol.

[7] Theparee T, Das S, Thomson RB Jr (2018) Total laboratory automation and matrix-assisted laser desorption ionization-time of flight mass spectrometry improve turnaround times in the clinical microbiology laboratory: a retrospective analysis. J Clin Microbiol 56(1):e01242–17 10.1128/JCM.01242-17 PMC5744220 29118171

[8] Verroken A, Defourny L, le Polain de Waroux O, Belkhir L, Laterre PF, Delmee M, Glupczynski Y (2016). Clinical impact of MALDI-TOF MS identification and rapid susceptibility testing on adequate antimicrobial treatment in sepsis with positive blood cultures. Plos One.

[9] Angeletti S, Dicuonzo G, D'Agostino A, Avola A, Crea F, Palazzo C, Dedej E, De Florio L (2015). Turnaround time of positive blood cultures after the introduction of matrix-assisted laser desorption-ionization time-of-flight mass spectrometry. New Microbiol.

[10] Osthoff M, Gurtler N, Bassetti S, Balestra G, Marsch S, Pargger H, Weisser M, Egli A (2017). Impact of MALDI-TOF-MS-based identification directly from positive blood cultures on patient management: a controlled clinical trial. Clin Microbiol Infect.

[11] Thomson RB, McElvania E (2019). Total laboratory automation: what is gained, what is lost, and who can afford it?. Clin Lab Med.

[12] Bailey AL, Ledeboer N, Burnham CD (2019). Clinical microbiology is growing up: the total laboratory automation revolution. Clin Chem.

[13] Cherkaoui A, Renzi G, Vuilleumier N, Schrenzel J (2019). Copan WASPLab automation significantly reduces incubation times and allows earlier culture readings. Clin Microbiol Infect.

[14] Cherkaoui A, Schrenzel J (2022). Total laboratory automation for rapid detection and identification of microorganisms and their antimicrobial resistance profiles. Front Cell Infect Microbiol.

[15] Lau AF (2021). Matrix-assisted laser desorption ionization time-of-flight for fungal identification. Clin Lab Med.

[16] Kostrzewa M, Nagy E, Schrottner P, Pranada AB (2019). How MALDI-TOF mass spectrometry can aid the diagnosis of hard-to-identify pathogenic bacteria - the rare and the unknown. Expert Rev Mol Diagn.

[17] Jang KS, Kim YH (2018). Rapid and robust MALDI-TOF MS techniques for microbial identification: a brief overview of their diverse applications. J Microbiol.

[18] Scott JS, Sterling SA, To H, Seals SR, Jones AE (2016). Diagnostic performance of matrix-assisted laser desorption ionisation time-of-flight mass spectrometry in blood bacterial infections: a systematic review and meta-analysis. Infect Dis (Lond).

[19] Tan KE, Ellis BC, Lee R, Stamper PD, Zhang SX, Carroll KC (2012). Prospective evaluation of a matrix-assisted laser desorption ionization-time of flight mass spectrometry system in a hospital clinical microbiology laboratory for identification of bacteria and yeasts: a bench-by-bench study for assessing the impact on time to identification and cost-effectiveness. J Clin Microbiol.

[20] Cherkaoui A, Renzi G, Azam N, Schorderet D, Vuilleumier N, Schrenzel J (2020). Rapid identification by MALDI-TOF/MS and antimicrobial disk diffusion susceptibility testing for positive blood cultures after a short incubation on the WASPLab. Eur J Clin Microbiol Infect Dis.

