# Supplementary material for: Diagnostic test accuracy of an automated device for the MALDI target preparation for microbial identification
Source: Eur J Clin Microbiol Infect Dis. 2022 Dec 5;42(2):153–9. doi: 10.1007/s10096-022-04531-3 (PMC9836989; doi:10.1007/s10096-022-04531-3)
Supplement: Supplementary file 1 — Supplementary file1 (PDF 1420 KB) [file 10096_2022_4531_MOESM1_ESM.pdf]

Diagnostic test accuracy of an automated device for the MALDI target preparation for microbial identification

Abdessalam Cherkaoui[1,2*], Arnaud Riat[1], Gesuele Renzi[1], Adrien Fischer[1], and Jacques Schrenzel[1,3]

[1]Bacteriology Laboratory, Division of Laboratory Medicine, Department of Diagnostics, Geneva University Hospitals, 4 rue Gabrielle-Perret-Gentil, 1205 Geneva, Switzerland
[2]Faculty of Medicine, Geneva, Switzerland
[3]Genomic Research Laboratory, Division of Infectious Diseases, Department of Medicine, Geneva University Hospitals and Faculty of Medicine, Geneva, Switzerland

Keywords: MALDI-TOF/MS, Automation, WASPLab®, Copan Colibrí ™

*__Corresponding author__: Abdessalam CHERKAOUI, PD - PhD - FAMH
Bacteriology Laboratory, Division of Laboratory Medicine, Department of Diagnostics, Geneva University Hospitals, 4 rue Gabrielle-Perret-Gentil, 1205 Geneva, Switzerland
E-mail address: abdessalam.cherkaoui@hcuge.ch

	Species (number of isolates analyzed)	Clinical isolates								
		1	2	3	4	5	6	7	8	9 to 20
**Enterobacteriales**	***Escherichia coli* (n=20)**									Range
	Colibri^{TM} Biotyper score	2.35	2.29	2.35	2.08	2.3	2.42	2.34	2.35	2.37 - 2.45
	Manual_ Biotyper score	2.43	2.45	2.44	2.46	2.5	2.31	2.41	2.42	2.39 - 2.54
	***Klebsiella pneumoniae* (n=20)**									
	Colibri^{TM} Biotyper score	2.29	2.33	2.34	2.35	2.33	2.36	2.31	2.23	2.31 - 2.47
	Manual_ Biotyper score	2.35	2.33	2.2	2.36	2.4	2.45	2.44	2.38	2.23 - 2.48
	***Citrobacter koseri* (n=20)**									
	Colibri^{TM} Biotyper score	2.51	2.56	2.57	2.61	2.58	2.47	2.58	2.51	2.44 - 2.61
	Manual_ Biotyper score	2.38	2.41	2.47	2.31	2.44	2.51	2.51	2.46	2.30 - 2.56
	***Klebsiella oxytoca* (n=20)**									
	Colibri^{TM} Biotyper score	2.35	2.4	2.34	2.39	2.4	2.44	2.41	2.39	2.41 - 2.52
	Manual_ Biotyper score	2.41	2.37	2.39	2.43	2.45	2.44	2.46	2.43	2.05 - 2.46
	***Citrobacter freundii* (n=20)**									
	Colibri^{TM} Biotyper score	2.45	2.52	2.46	2.48	2.52	2.52	2.53	2.51	2.27 - 2.55
	Manual_ Biotyper score	2.51	2.5	2.48	2.49	2.51	2.48	2.49	2.47	2.10 - 2.40
	***Proteus mirabilis* (n=20)**									
	Colibri^{TM} Biotyper score	2.47	2.42	2.48	2.41	2.31	2.39	2.53	2.42	2.43 - 2.56
	Manual_ Biotyper score	2.27	2.41	2.43	2.44	2.17	2.27	2.22	2.31	2.43 - 2.54
	***Serratia marcescens* (n=8)**									
	Colibri^{TM} Biotyper score	2.1	2.26	2.19	2.03	2.15	2.24	2.22	2.32	
	Manual_ Biotyper score	1.76	2.35	2.28	2.36	2.18	2.25	2.24	2.25	
	***Enterobacter cloacae* (n=8)**									
	Colibri^{TM} Biotyper score	2.17	2.15	2.2	2.17	2.16	2.13	2.03	2.17	
	Manual_ Biotyper score	2.18	2.18	2.28	2.26	2.23	2.22	2.22	2.15	
	***Morganella morganii* (n=8)**									
	Colibri^{TM} Biotyper score	2.63	2.65	2.67	2.58	2.59	2.5	2.64	2.54	
	Manual_ Biotyper score	2.64	2.67	2.67	2.56	2.61	2.67	2.68	2.31	
	***Klebsiella aerogenes* (n=8)**									
	Colibri^{TM} Biotyper score	2.38	2.48	2.4	2.42	2.28	2.43	2.41	2.45	
	Manual_ Biotyper score	2.41	2.39	2.5	2.07	2.29	2.24	2.43	2.21	
**Non-fermenting Gram-negative bacilli**	***Pseudomonas aeruginosa* (n=20)**									
	Colibri^{TM}_ Biotyper score	2.26	2.23	2.17	2.16	2.07	2.17	2.36	2.41	2.08 - 2.46
	Manual_ Biotyper score	2.39	2.46	2.44	2.43	2.51	2.4	2.38	2.37	2.23 - 2.47
	***Acinetobacter baumannii* (n=20)**									
	Colibri^{TM} Biotyper score	2.45	2.24	2.45	2.47	2.54	2.49	2.57	2.58	2.39 - 2.51
	Manual_ Biotyper score	2.53	2.5	2.53	2.55	2.44	2.46	2.51	2.58	2.25 - 2.47
	***Stenotrophomonas maltophilia* (n=20)**									
	Colibri^{TM} Biotyper score	2.06	2.11	2.05	2.11	1.97	2.22	2.11	2.22	2.3 - 2.48
	Manual_ Biotyper score	2.09	2.12	1.93	1.92	1.93	2.05	2.09	2.11	2.0 - 2.39
	***Pseudomonas monteilii* (n=8)**									
	Colibri^{TM} Biotyper score	1.99	1.87	1.92	1.75	1.98	1.84	1.9	2.05	
	Manual_ Biotyper score	2.3	2.28	2.27	2.3	2.27	2.32	2.22	2.18	
	***Pseudomonas putida* (n=8)**									
	Colibri^{TM} Biotyper score	1.85	1.95	1.85	2	2.09	2.03	2.15	2.05	
	Manual_ Biotyper score	2.13	2.09	2.09	2.05	2.12	2.13	2.07	2.05	
	***Achromobacter xylosoxidans* (n=8)**									
	Colibri^{TM} Biotyper score	1.98	2.03	1.7	1.99	1.97	2.08	1.98	1.53	
	Manual_ Biotyper score	1.81	1.96	1.84	1.91	1.75	1.95	1.75	1.81	
Haemophilus	***Haemophilus influenzae* (n=20)**									
	Colibri^{TM} Biotyper score	2.3	2.38	2.42	2.24	2.28	2.26	2.21	2.25	2.21 - 2.46
	Manual_ Biotyper score	2.25	2.3	2.1	2.2	2.24	2.38	2.24	2.21	2.19 - 2.35
Staphylococcus	***Staphylococcus aureus* (n=20)**									
	Colibri^{TM} Biotyper score	2.44	2.36	2.5	2.51	2.45	2.27	2.35	2.52	2.0 - 2.18
	Manual_ Biotyper score	2.47	2.3	2.2	2.32	2.48	2.23	2.44	2.12	2.01 - 2.46
	***Staphylococcus epidermidis* (n=20)**									
	Colibri^{TM} Biotyper score	2.12	2.14	2.14	2.11	2.14	2.11	2.18	2.1	2.0 - 2.13
	Manual_ Biotyper score	1.85	1.91	1.57	2.04	2.15	2.13	2.13	2.02	2.15 - 2.3
	***Staphylococcus hominis* (n=20)**									
	Colibri^{TM} Biotyper score	2.2	2.31	2.25	2.34	2.21	2.2	2.25	2.25	2.03 - 2.27
	Manual_ Biotyper score	2.04	2.03	1.89	1.96	1.94	2.15	2.07	2.17	2.11 - 2.31
	***Staphylococcus warneri* (n=8)**									
	Colibri^{TM} Biotyper score	2.08	2.13	2.11	2.16	1.99	2.08	1.9	2.11	
	Manual_ Biotyper score	2.16	2.14	2.17	2.12	1.98	1.89	1.99	1.88	
	***Staphylococcus lugdunensis* (n=8)**									
	Colibri^{TM} Biotyper score	2.12	2.09	2.07	1.88	1.7	2.01	1.7	2.09	
	Manual_ Biotyper score	1.84	1.67	1.82	1.58	2.1	1.84	2.08	2.11	
	***Staphylococcus haemolyticus* (n=8)**									
	Colibri^{TM} Biotyper score	2.34	2.4	2.34	2.36	2.13	2.25	2.39	2.3	
	Manual_ Biotyper score	2.02	2.08	1.98	2.01	2	2.28	2.2	2.11	
Enterococcus	***Enterococcus faecalis* (n=20)**									
	Colibri^{TM} Biotyper score	2	1.78	2.12	2.04	1.8	2.24	2.21	2.18	1.93 - 2.28
	Manual_ Biotyper score	2.28	2.48	2.48	2.38	2.45	1.95	2.26	2.17	1.92 - 2.4
	***Enterococcus faecium* (n=8)**									
	Colibri^{TM} Biotyper score	2.29	2.4	2.42	2.34	2.54	2.47	2.34	2.42	
	Manual_ Biotyper score	2.47	2.48	2.51	2.46	2.54	2.52	2.5	2.45	
Streptococcus	***Streptococcus constellatus* (n=8)**									
	Colibri^{TM} Biotyper score	2.13	2.12	2.02	2.4	2.18	2.22	1.98	2.4	
	Manual_ Biotyper score	2.02	1.86	2	1.9	2.21	2.16	1.94	2.2	
	***Streptococcus agalactiae* (n=8)**									
	Colibri^{TM} Biotyper score	2.24	2.33	2.23	2.31	2.27	2.22	2.35	2.37	
	Manual_ Biotyper score	2.3	2.32	2.27	2.36	2.42	2.4	2.33	2.29	
	***Streptococcus pyogenes* (n=8)**									
	Colibri^{TM} Biotyper score	2.29	2.51	2.31	2.34	2.41	2.41	2.36	2.34	
	Manual_ Biotyper score	2.23	2.31	2.34	2.25	2.34	2.31	2.41	2.36	
Gardnerella	***Gardnerella vaginalis* (n=8)**									
	Colibri^{TM} Biotyper score	2.09	2.14	2.19	2.11	2.05	2.07	2.03	2.07	
	Manual_ Biotyper score	2.11	2.04	2.2	2.09	1.94	1.98	2.03	2	
Corynebacterium	***Corynebacterium amycolatum* (n=8)**									
	Colibri^{TM} Biotyper score	2.32	2.12	2.23	2.29	1.9	2.06	2.05	2.08	
	Manual_ Biotyper score	1.92	1.67	1.63	1.89	1.87	1.89	1.92	1.89	
Candida	***Candida albicans* (n=8)**									
	Colibri^{TM}_ Biotyper score	2.33	2.15	2.29	2.16	2.2	2.23	2.14	2.23	
	Manual_ Biotyper score	2.07	2.23	1.99	1.91	2.11	2.02	2.13	2.11	

Table-S1: Biotyper score values of 416 (31 different species) non-duplicate strains. For each strain, the MALDI target was prepared manually and processed automatically by the Copan Colibri^{TM}

Species (number of isolates analyzed)	Clinical isolates				
	1	2	3	4	5
***S. epidermidis* (n=5)**					
Colibri^{TM}_ Biotyper score	2.21	2.22	2.19	2.21	2.3
Manual_ Biotyper score	2.24	2.21	2.26	2.19	2.29
***E. faecalis* (n=5)**					
Colibri^{TM}_ Biotyper score	2.23	2.3	2.47	2.56	2.39
Manual_ Biotyper score	2.32	2.36	2.4	2.37	2.39
***C. amycolatum* (n=5)**					
Colibri^{TM}_ Biotyper score	2.18	2.25	2.41	2.29	2.17
Manual_ Biotyper score	2.14	2.35	2.2	2.26	2.4
***C. kroppenstedtii* (n=5)**					
Colibri^{TM}_ Biotyper score	2.05	2.29	2.21	2.14	2.26
Manual_ Biotyper score	2.25	2.23	2.25	2.37	2.25
***G. vaginalis* (n=4)**					
Colibri^{TM}_ Biotyper score	2.11	2.33	2.28	2.55	
Manual_ Biotyper score	2.38	2.3	2.45	2.4	
***L. iners* (n=1)**					
Colibri^{TM}_ Biotyper score	2.36				
Manual_ Biotyper score	2.33				
***C. albicans* (n=5)**					
Colibri^{TM}_ Biotyper score	2.16	2.05	2.11	2.09	2.12
Manual_ Biotyper score	1.84	1.98	1.98	1.98	1.99

Table-S2: Biotyper score values of a further set of Gram positive bacteria and yeast isolates using the formic acid method by the two compared methods
